# Evaluating emotional distress and health-related quality of life in patients with heart failure and their family caregivers: Testing dyadic dynamics using the Actor-Partner Interdependence Model

**DOI:** 10.1371/journal.pone.0227129

**Published:** 2020-01-08

**Authors:** Patricia Thomson, Kate Howie, Stephen J. Leslie, Neil J. Angus, Federico Andreis, Robert Thomson, Andrea R. M. Mohan, Catherine Mondoa, Misook L. Chung

**Affiliations:** 1 Faculty of Health Sciences and Sport, University of Stirling, Stirling, United Kingdom; 2 Faculty of Natural Sciences, University of Stirling, Stirling, United Kingdom; 3 Cardiac Unit, Raigmore Hospital, NHS Highland, Inverness, Scotland, United Kingdom; 4 Centre for Health Science, School of Health, Social Care and Life Sciences, University of the Highlands and Islands, Inverness, Scotland, United Kingdom; 5 College of Medicine, Dentistry & Nursing, University of Glasgow, Glasgow, Scotland, United Kingdom; 6 Cardiology Unit, Forth Valley Royal Hospital, NHS Forth Valley, Larbert, Scotland, United Kingdom; 7 College of Nursing, University of Kentucky, Lexington, KY, United States of America; University of Lleida, SPAIN

## Abstract

**Purpose:**

1) To compare levels of emotional symptoms and health-related quality of life between patients with heart failure and their family caregivers; and 2) to examine whether patients’ and caregivers’ emotional symptoms were associated with their own, as well as their partner’s health-related quality of life.

**Method:**

In this cross-sectional study, 41 patients-caregiver dyads (78% male patients, aged 68.6 years; and 83% female caregivers, aged 65.8 years) completed all nine dimensions of the Brief Symptom Inventory and the Minnesota Living with Heart failure Questionnaire. Dyadic data were analysed for 6 sub-scales of the Brief Symptom Inventory, using the Actor–Partner Interdependence Model.

**Results:**

There were no statistically significant differences in emotional symptoms and health-related quality of life between patients with heart failure and their caregivers. Patients’ and caregivers’ emotional symptoms were associated with their own health-related quality of life. Caregivers’ anxiety, phobic anxiety, obsession-compulsion, depression and hostility negatively influenced their partner’s (i.e. the patient’s) health-related quality of life. There were no *partner effects* of patients’ emotional symptoms on the health-related quality of life of caregivers.

**Conclusions:**

The results of this study suggest that patients may be particularly vulnerable to the emotional distress, i.e. thoughts, impulses and actions of their caregivers. It may be possible to improve patients’ health-related quality of life by targeting specific detrimental emotional symptoms of caregivers.

## 1. Introduction

Heart failure is a progressive condition characterized by frequent hospitalisations and significant morbidity and mortality [1–3]. Heart failure has a prevalence of 1–2% in developed countries which increases with age [3]. Patients with heart failure report psychological distress and reduced quality of life (QoL) [4–6], related to symptom burden, episodes of decompensation and prolonged hospital admissions [7–8]. Patient anxiety and depression are common psychological problems in heart failure [9–10], with anxiety prevalence ranging from 6% to 72% [11], and depression prevalence ranging from 9% to 60% [12]. Greater psychological distress has been reported to be associated with poor health outcomes and poor self-care in patients with heart failure [13–18].

Caregivers’ health and emotional well-being can be negatively affected when patients have heart failure [13,19–24]. When caregivers are over-burdened or depressed, patients have more than a two times increased risk of hospitalization and mortality [25]. Higher caregiver strain is associated with greater patient symptoms and lower patient QoL [25–27]. Being supported by a caregiver is important and can improve patient outcomes, including their ability to perform self-care [13,20,28–30]. It is therefore essential to recognise emotional distress in caregivers and to offer appropriate support [20,21,26].

Caregivers of patients with depressive symptoms have higher levels of caregiver burden and report worse mental-health related QoL [23]. Caregiver burden may also negatively influence the patient and caregiver relationship [13,31], as a consequence of poor communication, causing tensions and distress among couples [32–34]. Good relationship quality has been shown to be positively associated with caregiver benefit and negatively associated with caregiver burden [31,35]. Although research has examined patient and caregiver relationships in heart failure [13,34], and health-related QoL in patient-caregiver dyads [13,19,28,30], relatively few studies have examined emotional symptoms and health-related QoL in heart failure patient and caregiver dyads [14,19,36]. Only one study was found that examined anxiety and depression in patient-caregiver dyads, using dyadic regression to determine how one person’s emotional symptoms impact on their own and their partners health-related QoL [37].

Similar to Chung et al. [37], depressive symptoms and anxiety were assessed using the BSI and health-related QoL was assessed using the MLHFQ, and dyadic data were analysed using the APIM with distinguishable dyad regression. Our research differed from the US study by Chung et al. [37] in that we recruited a UK sample of heart failure patients and their caregivers and we examined other emotional symptoms, in addition to depression and anxiety. This study extends the body of knowledge on the *actor* and *partner effects* of emotional symptoms on health-related QoL of heart failure patient-caregiver dyads, using the APIM. Previous research has mostly involved a single assessment of either patient or caregiver outcomes [4,8,10,11,14,19,21,26,38,39]. Such an individualized approach ignores the interdependency of beliefs or behaviours within the patient and caregiver relationship [40].

Because both patients and caregivers are affected by the patients’ health status, interactions in patient-caregiver dyads are inevitable. The relationship between patient and caregiver is non-independent. The Actor-Partner Interdependence Model (APIM), based on Interdependence theory, allows investigators to examine the inter-relatedness of variables in dyads [41]. It provides insights into dyadic interactions by taking both the individual and caregiver contribution into account in a single regression model. In the APIM, the association between a predictor (independent variable) and outcome (dependent variable) for members of a dyad is composed of two distinct parts: the *actor effect* is the impact of a person’s own predictor variable on his or her outcome. The *partner effect* is the impact of a person’s predictor variable on his/her dyadic partner’s outcome [41–43]. This study aimed: 1) to compare levels of emotional symptoms and health-related QoL between patients with heart failure and their family caregivers; and 2) to examine whether patients’ and caregivers’ emotional symptoms were associated with their own, as well as their caregiver’s health-related QoL. Based on previous literature we hypothesise that caregivers would have worse health-related QoL compared to patients [36, 37], and greater emotional distress [21, 37]. Also, that caregivers’ emotional symptoms would impact on their own, and their partner’s (i.e. the patient’s) health-related QoL.

## 2. Method

### 2.1 Study design

This was a cross-sectional study of patients with heart failure and their family caregivers recruited from two community-based heart failure services in Scotland.

### 2.2. Setting and participants

Data were collected using a convenience sampling method between 2014 and 2015. Eligible patients were aged 40 years or over. The patients had a confirmed medical diagnosis of chronic heart failure for at least 3 months and they were stable (i.e. outpatients), on stable doses of heart failure medication. Spouses and partners (hereafter referred to as family caregivers or caregivers) were recruited providing they lived in the same household as the patient and were identified by them as their primary carer. Both patients and caregivers were excluded if there were any major co-morbidities, such as stroke or cancer, or psychological or communication limitations likely to affect their ability to consent.

### 2.3 Ethical considerations

This study was approved by the University of Stirling Ethics and Research Committee and the National Research and Ethics Committee (NRES), North of Scotland (Rec ref 13/NS/0013 (IRAS project ID: 118000).

### 2.4 Procedure

Patients and their caregivers were recruited on their visit to the heart failure clinic. Study information and consent forms were distributed by the heart failure nurse specialists, in accordance with the inclusion and exclusion criteria. The patient-caregiver pairs (i.e. dyads) were informed of the aims of the study and of the right to withdraw from participation, and they were assured of confidentiality and anonymity. After receipt of the signed consent forms the researcher posted questionnaire packs to the participant’s home address or provided a link to the Bristol on-line survey for completion, depending on their preference. The dyads were asked to complete the questionnaires without discussing answers with each other. Completed questionnaires were returned to the researcher by post or email. A reminder letter was sent after 2 weeks.

### 2.5 Instruments

#### Brief symptom inventory

Emotional symptoms were assessed using the 53-item Brief Symptom Inventory (BSI) [44]. This self-administered tool assesses psychological distress with respect to nine primary symptom dimensions: somatisation (i.e., greater distress arising from perspectives of bodily dysfunction, 7 items); obsession-compulsion (i.e., thoughts, impulses and actions that are unremitting and irresistible, 6 items); interpersonal sensitivity (i.e., self-depreciation, self-doubt, discomfort during interpersonal interactions, 4 items); depression (i.e., dysphoric mood and affect, 6 items); anxiety (i.e., feelings of apprehension and panic, 6 items); hostility (i.e., negative affect state of anger, 5 items); phobic anxiety (i.e., persistent fear response, 5 items); paranoid ideation (i.e., projective thoughts, hostility, suspiciousness, 5 items); and psychoticism (i.e., withdrawal, interpersonal alienation and psychosis, 5 items). Four additional items (i.e., poor appetite, trouble falling asleep, thoughts of death or dying, and feelings of guilt) were not used in the study because they ‘load on several dimensions of the BSI but are not univocal to any of them; they are not scored collectively and do not form a dimension’ [45, p.10]. All items on the BSI were rated on a 5-point Likert scale ranging from 0 (not at all) to 4 (extremely). For each dimension, the scores were summed and means obtained. Higher scores indicate higher levels of emotional distress. Raw scores were converted to standardized T scores for comparison with healthy populations [45].

Construct validity of the 9 factor structure BSI has been reported in research [45–51], across different populations. Although Derogatis and Melisaratos [52] proposed the 9 factors, they acknowledge there were minor differences between the empirical factor structure and those hypothesised, although there was more agreement than disagreement between the two. Other studies have reported varying numbers of BSI factor structures, ranging from 1 to 8 factors [53–56].

Cardiovascular studies have used the BSI [57,58], but limited or no information has been provided on psychometric testing. Kushner et al. [59] used discriminant function analysis in a study of panic disorders in cardiology patients that revealed 1 item, i.e. anxiety. Khalil et al. [60] identified 2 BSI factors (depression and anxiety) in patients with heart failure and normal renal function. A valid 3 factor BSI sub-scale was identified by Roser [61] in a study examining the biobehavioral influences of anxiety, depression and hostility on health-related outcomes in patients with heart failure. Other cardiovascular studies have employed 1 or 2 BSI factors (anxiety, depression) with patients [62–67], and with patients and/or caregivers [68–70], but construct validity was not tested.

Heart failure studies have reported Cronbach’s alpha for BSI anxiety that range from 0.74 to 0.86 for patients [6, 10, 21, 37], and 0.87 for spouses [37]. Cronbach’s alpha for BSI depression have been reported as 0.92 for patients and 0.83 for spouses [37]. In this study Cronbach’s alpha for the 9 BSI sub-scales ranged from 0.72 to 0.91 for patients and from 0.65 to 0.94 for caregivers. **Table 1** shows comparison of the Cronbach’s alpha with those of Derogatis and Melisaratos [52] in their introductory report for the BSI [44].

**Table 1:** Comparison of internal consistencies for the 9 symptom dimensions of the BSI.

	No of items	Study Patients (N = 41)	Study Caregivers (N = 41)	Derogatis & Melisaratos 1983 (N = 719)
Internal consistency (α)				
Symptom dimension				
Somatisation	7	0.83	0.85	0.68
Obsession-compulsion	6	0.90	0.94	0.85
Interpersonal sensitivity	4	0.82	0.79	0.85
Depression	6	0.89	0.93	0.84
Anxiety	6	0.90	0.91	0.79
Hostility	5	0.79	0.65	0.81
Phobic anxiety	5	0.91	0.82	0.91
Paranoid ideation	5	0.72	0.65	0.79
Psychoticism	5	0.72	0.86	0.78

#### Minnesota living with heart failure

The 21-item self-administered Minnesota Living with Heart Failure Questionnaire (MLHFQ) [71,72], was used to assess patients’ and caregivers’ health-related QoL, defined as individual’s perceptions of the effects of heart failure and its treatment on their daily lives [73]. Specifically, the MLHFQ measures the effects of symptoms, functional limitations and psychological reactions commonly associated with heart failure or its treatment on the individual’s QoL [74]. Respondents rated items on a six-point Likert scale from 0 (no effect) to 5 (very much), which were summed to obtain a total score ranging from 0 to 105, with higher scores indicating poorer health-related QoL. The unidimensionality of the MLHFQ (total score) has been supported in research [75], and internal reliability in studies of heart failure patients [36,38,39,71,76–79], and patient-caregiver dyads [37].

Slight modification was made to the MLHFQ to fit the context relevant to caregivers [37]. This involved rewording of the introduction for caregivers to indicate to them how to answer the questions appropriately for themselves and not the patient. Another two items were modified to fit in caregiver’s view; item 16 ‘giving you side effects from medications?’ was replaced with ‘giving you less time to take care of your own physical health?’ and Item 17 ‘making you feel you are a burden to your family or friends?’ was replaced to ‘feeling burdened by your family member?’ In previous studies, the internal consistency of the MLHFQ has been demonstrated with Cronbach’s alpha of 0.93 for patients and 0.95 for caregivers [37]. In the present study, the Cronbach’s alpha was 0.94 for patients and 0.96 for caregivers.

#### Sociodemographic and clinical characteristics

Data on age, gender, employment status and education were collected by questionnaire. Occupation was identified in accordance with the National Statistics Office [79]. Social deprivation was identified using an index that takes account of income and residential postcode [80], with categories ranging from 1 (most affluent) to 7 (most deprived). Left ventricular ejection fraction (LVEF), aetiology, co-morbidity, i.e. hypertension, diabetes, depression and current medications use were identified from patients’ clinical records.

### 2.6 Statistical analysis

Comparison of socio-demographics, emotional symptoms and health-related QoL between patients and caregivers were computed using paired sample *t*-tests, or chi-square statistics. Spearman's Rank correlation coefficient was used to identify the relationship between each of the 9 BSI dimensions and health-related QoL. When the BSI items were very strongly inter-correlated with each other (i.e. > 0.8) they were omitted from further (i.e. dyadic) analysis as they were deemed to be too closely related constructs. The Actor-Partner Interdependence Model (APIM) regression for distinguishable dyads was used to examine the impact of patients’ and caregivers’ emotional symptoms (independent variables) on their own, as well as their partner’s health-related QoL (dependent variable) [41,43]. The *actor effect* is the effect of an individual’s characteristics (i.e., emotional symptoms) on their own health-related QoL. The *partner effect* refers to the effect of an individual’s characteristics (i.e., emotional symptoms) on their partner’s health-related QoL [41–42]. We used the online app developed by Stas et al. [81], ‘which automatically performs the statistical analyses associated with the APIM, using lavaan. Because structural equation model (SEM) techniques are used to fit the APIM, the app is called APIM_SEM’ [p.103].

Separate APIM models were computed; health-related QoL was regressed for each of the dimensions of the BSI, as appropriate; with p < 0.05 indicating statistical significance.

## 3. Results

### 3.1 Characteristics of the participants

Out of the 53 patients who consented to participate in the study on their visit to the heart failure clinic, 6 were eliminated because they did not have a caregiver, or the caregiver did not consent to participate in the study. Six patient-caregiver dyads consented to participate but failed to return the questionnaires. The data analysis was based on 41 patient-caregiver dyads. The socio-demographics, clinical history, emotional symptoms and health-related QoL of the participants are presented in **Table 2.** Most patients were men (78%) and most caregivers were women (83%), which is often typical in studies of patients and caregivers [82]. Patients mean age was 68.6 years (SD = 10.8) and caregivers mean age was 65.8 years (SD = 10.6). Half of the patients were in NYHA class I or II; and 66% had a left ventricular ejection fraction of less than 29%. Only 12% of patients were on antidepressants.

**Table 2:** Patients and family caregivers characteristics (n = 41).

Characteristics	Patients	Caregivers	p
Age in years (median, range)	70.0 (40–86)	66.0 (43–84)	0.063
Males	32 (78%)	7 (12%)	0.327
Employment			
Employed	10 (24.4%)	13 (31.7%)	
Unemployed or retired	31 (75.6%)	28 (68.3%)	0.005*
Education in years (median, range)	13.0 (5–20)	11.00 (5–24)	0.494
Social deprivation (SIMD)			
SIMD 1–2	8 (23%)	-	-
SIMD 3–5	27 (77%)	-	-
Left ventricular ejection fraction			
> 50%	5 (12.2%)	-	-
30–49% (moderate impairment)	9 (22.0%)	-	-
< 29% (severe impairment)	27 (65.8%)	-	-
New York Heart Association (NYHA)			
Class 1/11	20 (48.8%)	-	-
Class 111/1V	18 (43.9%)	-	-
Missing	3 (7.3%)	-	-
Aetiology			
Ischaemic	12 (29.3%)	-	-
Idiopathic	2 (4.9%)	-	-
Hypertension	5 (12.2%)	-	-
Other /missing	22 (53.6%)	-	-
History of hypertension	11 (26.8%)	-	-
History of diabetes mellitus	3 (7.3%)	-	-
Medications			
ACE	24 (58.5%)	-	-
Beta blocker	30 (73.1%)	-	-
Diuretics	25 (61.9%)	-	-
Antidepressants	5 (12.1%)	-	-
Brief Symptom Inventory			
Somatization,	1.02 (0.7)	0.56 (0.8)	0.017*
Obsession-compulsion	1.02 (0.9)	0.89 (1.0)	0.583
Interpersonal sensitivity	0.51 (0.7)	0.40 (0.6)	0.506
Depression	0.62 (0.8)	0.53 (0.8)	0.597
Anxiety	0.66 (0.8)	0.65 (0.9)	0.987
Hostility	0.44 (0.5)	0.44 (0.5)	0.981
Phobic anxiety	0.64 (0.9)	0.34 (0.6)	0.103
Paranoid ideation	0.33 (0.6)	0.35 (0.5)	0.877
Psychoticism	0.39 (0.6)	0.31 (0.6)	0.522
MLHFQ			
Total score	38.27 (22.9)	30.34 (25.0)	0.100

SIMD, Scottish Index of Multiple Deprivation; ACE, angiotensin converting enzyme inhibitor; MLHFQ, Minnesota Living with Heart Failure Questionnaire; p < 0.05*

Twenty patients (49%) and 6 caregivers (15%) completed the Bristol on-line survey and 5 patients (12%) and 10 caregivers (24%) completed paper copies of the questionnaires. The instructions/stems included in the questionnaires were replicated at the start of the on-line survey. Our brief evaluation of the on-line survey (five open questions) suggested that it was clear and easy to use and the content clear. The participants who opted to complete the paper copies of the questionnaires had ‘none’ or ‘very limited’ computer and internet skills.

### 3.2 Comparisons of emotional symptoms and health-related quality of life between patients and caregivers

There were no statistically significant differences between the patients’ and caregivers’ emotional symptoms, except for somatisation **(Table 3).** Patients had higher scores for somatization (1.02 vs. 0.56, p = 0.017), indicating their greater distress arising from perspectives of bodily dysfunction. Health-related QoL were not statistically significantly different between the patients and caregivers (38.27 vs 30.34, p = 0.1) (Table 3).

**Table 3:** Patients and caregivers’ emotional symptoms and health-related QoL (n = 41 dyads).

Characteristics	Patients	Caregivers		
	Means (SD)		Paired t test	*p*
Somatization, BSI	1.02 (0.7)	0.56 (0.8)	2.49	0.017*
Obsession-compulsion, BSI	1.02 (0.9)	0.89 (1.0)	0.55	0.583
Interpersonal sensitivity, BSI	0.51 (0.7)	0.40 (0.6)	0.67	0.506
Depression, BSI	0.62 (0.8)	0.53 (0.8)	0.53	0.597
Anxiety, BSI	0.66 (0.8)	0.65 (0.9)	0.02	0.987
Hostility, BSI	0.44 (0.5)	0.44 (0.5)	-0.02	0.981
Phobic anxiety, BSI	0.64 (0.9)	0.34 (0.6)	1.67	0.103
Paranoid ideation, BSI	0.33 (0.6)	0.35 (0.5)	-0.16	0.877
Psychoticism, BSI	0.39 (0.6)	0.31 (0.6)	0.65	0.522
HQoL, MLHFQ	38.27 (22.9)	30.34 (25.0)	1.68	0.100

BSI, Brief Symptom Inventory; HQoL, health-related quality of life; MLHFQ, Minnesota Living with Heart Failure Questionnaire

*p < 0.05

### 3.3 Correlations among the BSI dimensions and health-related quality of life

**Table 4** shows the relationship between each of the 9 BSI dimensions and health-related QoL. Patient hostility was moderately negatively correlated with their own health-related QoL. Several caregivers’ emotional symptoms were weakly positively correlated with the patients’ health-related QoL. Caregivers’ health-related QoL was moderately positively correlated with several of their own BSI dimensions. Somatisation, interpersonal sensitivity and psychoticism were very strongly intercorrelated (i.e. > 0.8) with other BSI items, indicating they were closely related constructs. **Table 4** shows additional information on the intercorrelations between the variables.

**Table 4:** Correlations coefficients among British Symptom Inventory dimensions and quality of life (n = 41 dyads).

	1	2	3	4	5	6	7	8	9	10	11	12	13	14	15	16	17	18	19	20
**Patients**																				
1. QoL	1																			
2. SOM	-.21	1																		
3. OC	-.29	.83**	1																	
4. IS	-.22	.63**	.70**	1																
5. DEP	-.21	.59**	.62**	.86**	1															
6. ANX	-.21	.77**	.75**	.77**	.78**	1														
7. HOS	-42**	.53**	.66**	.66**	.64**	.67**	1													
8. PHOB	-.21	.63**	.72**	.73**	.65**	.68**	.51**	1												
9. PAR	-.20	.46**	.59**	.70**	.67**	.58**	.61**	.46**	1											
10. PSY	-.13	.58**	.75**	.81**	.82**	.68**	.60**	.69**	.72**	1										
**Caregivers**																				
11. QoL	.26	-.25	-.20	-.12	-.14	-.26	-.14	-.32*	-.08	.02	1									
12.SOM	.21	-.22	-.18	-.05	-.11	-.22	-.12	-.20	-.19	-.01	.58*	1								
13. OC	.37*	-.20	-.18	-.06	-.07	-.12	-.06	-.23	.02	.01	.70**	.69**	1							
14. IS	.29	-.18	-.16	-.24	-.15	-.25	-.08	-.16	-.23	.01	.51**	.68**	.51*	1						
15. DEP	.31*	-.04	-.05	.02	.01	-.06	-.14	-.16	.01	.11	.71**	.76**	.71**	.62**	1					
16. ANX	.40**	-.12	-.14	-.05	.10	-.03	-.05	.25	.04	.12	.74**	.69**	.79**	.54**	.78**	1				
17. HOS	.31*	-.24	-.21	-.18	-.02	-.18	-.2	-.26	.03	-.03	.76**	.40**	.69**	.44**	.61**	.71**	1			
18.PHOB	.37*	.08	.08	.12	.17	.02	.09	-.1	.15	.27	.47**	.64**	.58**	.59**	.71**	.73**	.40**	1		
19. PAR	.14	-.16	-.06	-.17	-.04	-.27	-.11	-.21	-.02	.12	.62**	.50**	.58**	.66**	.63**	.57**	.61**	.47**	1	
20. PSY	.18	-.04	.03	-.01	0.10	-.09	.06	-.15	.22	.29	.56**	.47**	.74**	.53**	.61**	.60**	.57**	.60**	.71**	1

QoL, quality of life; SOM, somatization; OC, obsessive-compulsive; IS, interpersonal sensitivity; DEP, depression; ANX, anxiety; HOS, hostility; PHOB, phobic anxiety; PAR, paranoid ideation; PSY, psychoticism

** p < .001

*p < .005

### 3.4 Impact of emotional distress on health-related quality of life

Based on the results of the correlations dyadic analysis using the APIM was conducted for 6 emotional symptom dimensions (i.e. obsession-compulsion, depression, anxiety, hostility, phobic anxiety and paranoid ideation) and health-related QoL. Somatisation, interpersonal sensitivity and psychoticism they were omitted from the dyadic analysis as they were very strongly intercorrelated with other BSI items **(Table 5).** The results for the 6 emotional distress dimensions (BSI) and health-related QoL are presented in **Table 5.**

**Table 5:** Actor and partner effects of emotional symptoms on health-related QoL using the APIM.

Emotional symptoms (BSI)	Patients			Caregivers		
	Beta	*t*	*p*	Beta	*t*	*p*
Anxiety						
Actor effect	9.657	3.53	0.006	20.300	2.90	<0.001
Partner effect	3.162	3.16	0.317	11.532	3.25	<0.001
Phobic anxiety						
Actor effect	6.892	3.18	0.030	21.047	5.59	<0.001
Partner effect	1.327	3.48	0.703	14.271	5.10	0.005
Paranoid ideation						
Actor effect	16.303	5.55	0.003	21.362	5.15	<0.001
Partner effect	4.855	5.70	0.394	7.220	5.02	0.150
Obsession-compulsion						
Actor effect	9.008	3.01	0.003	13.426	3.09	<0.001
Partner effect	0.006	3.82	0.999	11.272	2.57	<0.001
Depression						
Actor effect	9.936	3.87	0.010	20.577	3.65	<0.001
Partner effect	2.160	3.64	0.553	9.081	3.88	0.019
Hostility						
Actor effect	10.927	6.08	0.072	34.518	4.78	<0.001
Partner effect	9.126	4.67	0.051	13.251	6.22	0.033

APIM, Actor Partner Interdependence Model; BSI, Brief Symptom Inventory Model–fitted using APIM: Distinguishable Dyads Using lavaan [Stas et al 2018].

Among the 6 emotional symptoms, there were 5 statistically significant *actor effects* of emotional symptoms on health-related QoL of patients, indicating those who reported higher levels of emotional distress (anxiety, phobic anxiety, paranoid ideation, obsession-compulsion and depression) had poorer health-related QoL (higher scores on the MLHFQ indicate worse health-related QoL). For caregivers, among the 6 emotional symptoms, there were statistically significant *actor effects* of all emotional symptoms on health-related QoL, indicating caregivers who reported higher levels of emotional distress (anxiety, phobic anxiety, paranoid ideation, obsession-compulsion, depression and hostility) had poorer health-related QoL.

**Fig 1:**
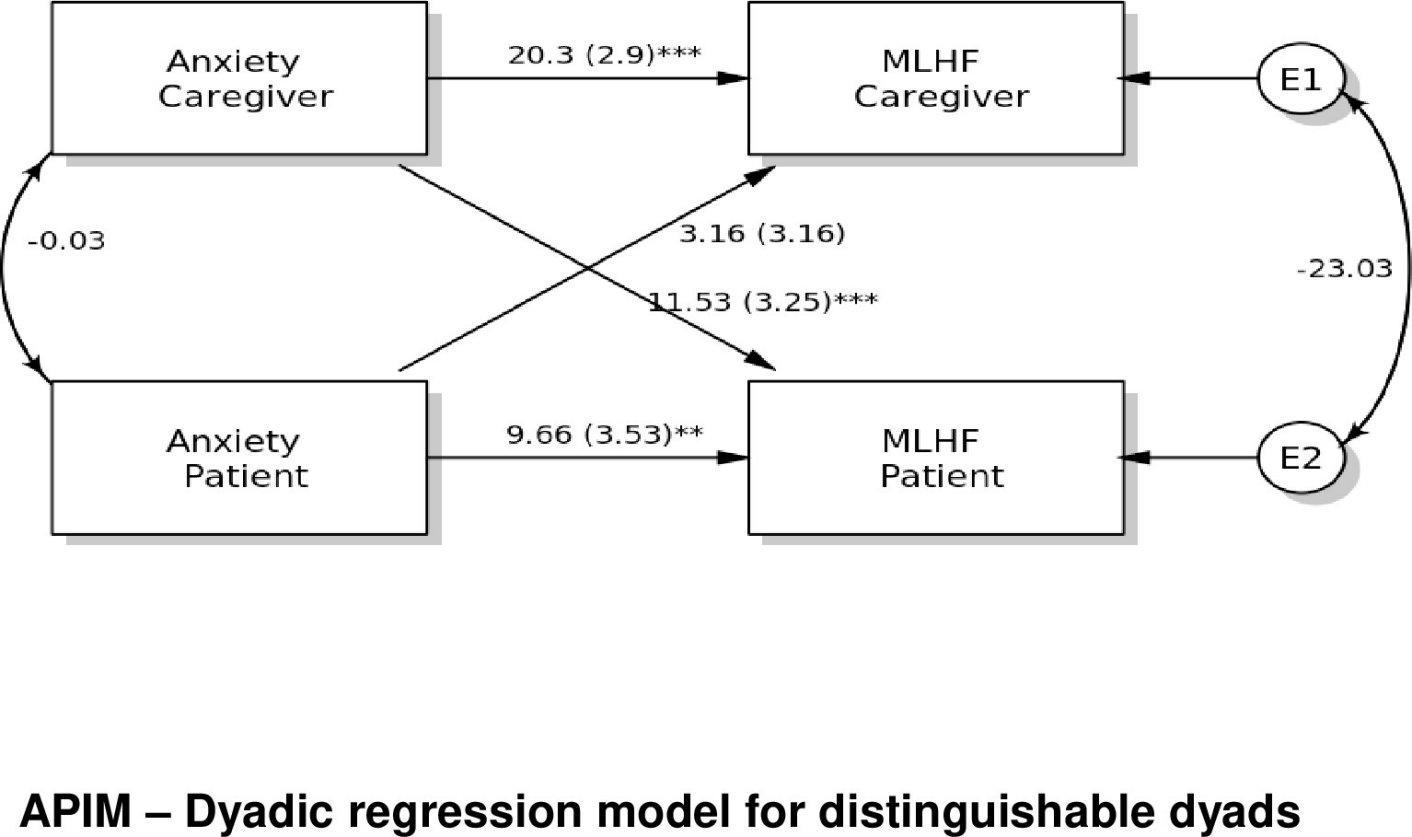
Results for the *actor* and *partner* effects of patient’s anxiety and caregiver’s anxiety on health-related quality of life using the MLHFQ, Minnesota Living with Heart Failure Questionnaire; BSI, Brief Symptom Inventory; APIM: Actor-Partner Interdependence Model. * p < .05; ** p < .01; *** p < .001.

There were statistically significant *partner effects* of caregivers’ emotional symptoms on the health related QoL of patients (**Table 5**). In contrast, there were no statistically significant *partner effects* of patients’ emotional symptoms on the health-related QoL of caregivers, although there was a trend for hostility (p = 0.051). This indicated that patient’s greater hostility (i.e., negative affect state of anger) may negatively influence the caregiver’s health-related Qol. Among caregivers’ emotional symptoms, there were 5 statistically significant *partner effects*. The caregiver’s anxiety, phobic anxiety, obsession-compulsion, depression and hostility negatively influenced their partner’s (i.e. patient’s) health-related Qol. Caregivers’ higher levels of these emotional symptoms were associated with the patients’ poorer health-related QoL. **Fig 1** presents the APIM model results showing the *actor effects* of patients’ anxiety and caregivers’ anxiety on their own health-related QoL, and a *partner effect* of caregiver's anxiety on the health-related Qol of the patient.

## 4. Discussion

This is the first study to apply a different analysis method with the aim of understanding relationships between patient and caregiver symptoms of psychopathology and distress.

Similar to Chung et al. [37], depressive symptoms and anxiety were assessed using the BSI and health-related QoL was assessed using the MLHFQ. In the same way, dyadic data were analysed using the APIM, with distinguishable dyad regression. Our research differed from the US study by Chung et al. [37], in that we recruited a UK sample of heart failure patients and their caregivers and we examined additional emotional symptoms besides depression and anxiety. This study extends the body of knowledge on the *actor* and *partner effects* of emotional symptoms on health-related QoL of heart failure patient-caregiver dyads, using the APIM. Previous research into emotional symptoms and health-related QoL have often been examined in patients and caregivers separately [63,83], or investigators have used a different type of statistical analysis in examining the patient-caregiver dyads [14,19,36]. Both patients with heart failure and their caregivers experienced substantial emotional distress. This high prevalence of emotional distress is consistent with other studies’ findings [4,5,9,10,19]. Analysis of all 9 BSI sub-scales revealed the patients’ and caregivers’ scores were not statistically significantly different, except for somatisation (i.e., greater distress arising from perspectives of bodily dysfunction), which was not unexpectedly higher in patients. This finding is consistent with earlier studies that found patient and caregiver congruence in mental health and wellbeing [34,84,85]. Compared to previous research [46,47], the patients’ somatisation and obsession-compulsion scores were high, indicating greater emotional distress arising from perspectives of bodily dysfunction, and thoughts, impulses and actions that were unremitting and irresistible. The other sub-dimensions of the BSI also scored highly when compared to British community norms [46,47], and US individuals (non-patients) [45].

Both patients with heart failure and their caregivers experienced similar levels of depressive symptoms and anxiety, which is comparable with other studies findings [4,8,11,12,19,37, 63,86]. Our results differ from some prior research [87], but concur with a previous study finding that suggest both patients and caregivers have similar depressive symptoms [88]. Our patients’ anxiety scores were lower than those found in previous studies [4,10,11,63], but caregivers’ anxiety scores were higher [21,37]. With respect to prevalence figures, 20 patients (48.7%) in this study reported depressive symptoms and 16 patients (39.0%) experienced anxiety. It is difficult to compare these figures because those cited in the literature are wide ranging. For example, Rutledge et al. [12] identify patient depression prevalence figures as ranging from 9% to 60%, and Easton et al (11] report anxiety prevalence figures ranging from 6.3% to 72.3%.

It is also known that 23 to 47% of caregivers of patients with heart failure experience mild to moderate depressive symptoms [87,88], and 45% of caregivers of patients with end-stage heart failure had depression, and as many as 50% of caregivers are anxious [73]. Our findings are consistent with previous research in identifying a significant a number of caregivers of heart failure patients experience depressive symptoms i.e. 17 (41.4%) caregivers had depression and 17 (41.4%) caregivers had anxiety levels that exceed those reported in healthy populations [44,45].

Overall, the non-significant results for differences in depressive symptoms and anxiety between the patients and caregivers suggest the emotional aspects of dealing with heart failure may affect the caregivers as much as their partners who have the illness [37]. One explanation for this similarity may lie with its links to the theory of emotional contagion which suggests that emotions are easily transferred to another person when two individuals are in an intimate interpersonal relationship [89,90].

We found that patients with heart failure and their caregivers’ health-related QoL (MLHFQ) scores were not statistically significantly different. The results for patients are consistent with prior research [37,38], but contrary to some other studies that found higher scores on the MLHFQ [4,36,39], indicating poorer health-related QoL. Our caregivers scores were low compared to prior research [13,36], indicating better health-related QoL. Overall, our results are in broad agreement with Chung et al. [37], revealing poorer health-related QoL in both patients and caregivers. To date, few studies have reported on caregivers’ health-related QoL, as measured by the MLHFQ.

In this dyadic analysis, the patients’ and caregivers’ emotional symptoms mostly influenced their own health-related QoL (i.e. *actor effects*). Our findings are consistent with Chung et al. [37] who found that depressive symptoms and anxiety exhibited both patient and caregiver *actor effects* on health-related QoL. Other studies have identified that patients’ emotional symptoms were related to their own poorer health-related QoL [6,10,19,38]. Similarly, caregivers’ emotional symptoms have been linked to their poorer health outcomes [13,19,21,24,87,91].

In addition, the dyadic analysis revealed important *partner effects* of emotional symptoms on the health-related QoL of patients. The caregiver’s anxiety, phobic anxiety, obsession-compulsion, depression and hostility negatively influenced their partner’s (i.e. the patient’s) health-related Qol. Our findings are consistent with Chung et al. [37] who identified that caregiver’s anxiety and depression had a *partner effect* on the patient’s health-related QoL, influencing it in a negative way. No previous APIM studies were identified for comparison of the results for phobic anxiety, obsession-compulsion and hostility. Previous research has highlighted that higher caregiver strain is associated with greater patient symptoms and lower patient QoL [25–27]. Other studies have found that if the caregiver is emotionally distressed this can affect their ability to support the patient [21,32,87,88], including their ability to perform self-care [13,20,29]. Further, Retrum et al. [34] identified that much of the tension and distress among dyads relates to conflicting views about how emotions should be dealt with or expressed. Several studies have reported on the health consequences of partner distress in coping with heart failure [21,22,24,92]. With respect to paranoid ideation there was no statistically significant dyadic effect i.e. the caregiver’s paranoid ideation did not impact their partner’s (i.e. the patient’s) health-related QoL. Possible explanations for this may be that paranoid ideation (i.e. projective thoughts, hostility, suspiciousness) is more to do with the patient themselves and reflective of more malignant emotional symptoms. Caregivers may be less likely to experience this emotional symptom and so not exhibit a *partner effect* on the health-related QoL of patients.

The dyadic analysis revealed no statistically significant *partner effects* of the patient’s emotional symptoms on the health-related QoL of caregivers. Patient’s emotional symptoms did not appear to significantly influence their partner’s (i.e. caregiver’s) health-related QoL. Our findings are consistent with Chung et al. [37] who found patients’ depressive symptoms and anxiety did not impact caregivers’ health-related QoL. There was a trend in the study for patient’s greater hostility (i.e., negative affect state of anger) to negatively influence the caregiver’s health-related QoL. No dyadic studies were found for comparison of our result although Moser et al. [83], identified that most patients with heart disease, including those with heart failure have depressive symptoms and that about 40% of these experience anxiety; and hostility was apparent in about half of these patients.

In summary, this study found the substantial impact of caregivers’ emotional symptoms such as anxiety, phobic anxiety, obsession-compulsion, depression and hostility on the health-related QoL of patients. This suggests that patients may be particularly vulnerable to the emotional distress i.e. thoughts, impulses and actions of their caregivers. This study extends previous research by demonstrating the effects of other caregivers’ emotional symptoms (apart from anxiety, depression and hostility) on the health-related QoL of patients with heart failure [37,87,88,91]. However, no relationship effect of emotional symptoms (i.e. from patient to caregiver) was apparent. The emotional symptoms were examined using an appropriate method of analysis that recognises the non-independence of patient and caregiver data.

## 5. Study limitations

There were limitations to this study. Firstly, this was a relatively small sample of patients and caregivers recruited from two centres. We have no information on the response rate, which could give rise to selection bias. Nevertheless, the study aimed to be representative of the wider UK heart failure population as patients were selected from a standard heart failure nurse led service. Study information and consent forms were distributed by the heart failure specialists in accordance with the inclusion and exclusion criteria. Although the average age of participants was lower than one might expect, older patients were less likely to have a dyadic caregiver. Secondly, length of marriage or cohabitation and marital quality of the respondents were not known and knowledge of these could be used to improve the model. Finally, the data were cross-sectional which meant that the direction of causality of associations could not be determined.

## 6. Implications for practice

There are several implications resulting from the findings of this study. Firstly, to improve heart failure patients’ health-related QoL emotional symptoms should be routinely assessed in both patients and caregivers. Secondly, it is important to decipher which emotional symptoms have more influence on caregiver outcomes and to target these appropriately. Thirdly, intervention formats should be expanded to include caregivers and to address their thoughts, fears and behaviours. Many current interventions focus on improving depressive symptoms/anxiety and quality of life only for patients [93,94]. To date, few intervention studies in heart failure patient-caregiver dyads have been tested [7,14]. Further research is needed to explore the complexity of interpersonal relationships and dyadic effects in heart failure. Research which includes covariates in the model, i.e. age, gender, occupation, length of partnership could lead to a deeper insight. Research is also needed evaluating the relationship of emotional symptoms and health-related QoL over time, using the APIM.

## 7. Conclusions

This study found no statistically significant differences in the levels of emotional symptoms and health-related QoL between heart failure patients and their caregivers. The study provides valuable insights regarding the *actor effect*s of the individual (i.e. patients and caregivers) emotional symptoms on their own health-related QoL. It identifies the *partner effect*s of caregiver’s emotional symptoms i.e. anxiety, phobic anxiety obsession-compulsion, depression and hostility on their partner’s (i.e. the patient’s) health-related QoL. This suggests the patient may be particularly vulnerable to the emotional distress i.e. thoughts, impulses and actions of their caregiver. If the caregiver is emotionally distressed this can affect their ability to support the patient. It may be possible to improve patients’ health-related QoL by targeting specific detrimental emotional symptoms of caregivers.

## Supporting information

S1 Dataset (SAV)

## References

[1] Chung ML, Lennie TA, Dekker RL, Wu J-R, Moser DK. Depressive symptoms and poor social support have a synergistic effect on event-free survival in patients with heart failure. Heart Lung. 2011;40(6): 492–501. 10.1016/j.hrtlng.2010.08.001 21453972 PMC3129423

[2] Schwarz KA, Elman CS. Identification of factors predictive of hospital readmissions for patients with heart failure. Heart Lung. 2003;32(2): 88–99. 10.1067/mhl.2003.15 12734531

[3] Ponikowski P, Voors AA, Anker SD, Bueno H, Cleland JGF, Coats AJS, et al ESC Guidelines for the diagnosis and treatment of acute and chronic heart failure: The Task Force for the diagnosis and treatment of acute and chronic heart failure of the European Society of Cardiology. Eur J Heart Failure. 2016;18: 891–975. 10.1002/ejhf.592 27207191

[4] Banerjee T, Lee KS, Browning SR, Hopenhayn C, Westneat S, Biddle MJ, et al Limited association between perceived control and health-related quality of life in patients with heart failure. J Cardiovasc Nurs. 2014;29(3): 227–31. 10.1097/JCN.0b013e31828b2b23 23507705 PMC3701030

[5] Joen YH, Kraus SG, Jowsey T, Glasgow NJ. The experience of living with chronic heart failure: a narrative review of qualitative studies. BMC Health Serv Res. 2010;10: 77 10.1186/1472-6963-10-77 20331904 PMC2851714

[6] De Jong MMJ, Moser DK, Chung ML. Predictors of health status for health failure patients. Prog Cardiovasc Nurs. 2005; 20: 155–62. 10.1111/j.0889-7204.2005.04649.x 16276138

[7] Liljeroos M, Agren S, Jaarsma T, Arestedt K, Stromberg A. Long term follow-up after a randomised integrated educational and psychosocial intervention in patient-partner dyads affected by heart failure. PLOS ONE. 2015 9 25 10.1371/journal.pone.0138058 26406475 PMC4583392

[8] Auld JP, Mudd JO, Gelow JM, Lyons KS, Hiatt SO, Lee CS. Patterns of heart failure symptom are associated with self-care behaviors over 6 months. Eur J Cardiovasc Nurs. 2018;17(6): 543–51. 10.1177/1474515118759074 29442523 PMC6067986

[9] Vongmany J, Hickman LD, Lewis J, Newton PJ, Phillips JN. Anxiety in chronic heart failure and the risk of increased hospitalisations and mortality: a systematic review. Eur J Cardiovasc Nurs. 2016;15(7): 478–85. 10.1177/1474515116635923 26912725

[10] De Jong MJ, Chung ML, Wu-JR. et al Linkages between anxiety and outcomes in heart failure. Heart Lung. 2011;40: 393–404. 10.1016/j.hrtlng.2011.02.002 21453974 PMC3149715

[11] Easton K, Coventry P, Lovell K, Carter LA, Deaton C. Prevalence and Measurement of Anxiety in samples of patients with heart failure. J Cardiovasc Nurs. 2016; 31(4): 367–79. 10.1097/JCN.0000000000000265 25930162 PMC4915759

[12] Rutledge T, Reis VA, Linke SE, Greenberg BH, Mills PJ. Depression in heart failure a meta-analytic review of prevalence, intervention effects, and associations with clinical outcomes. J Am Coll Cardiol. 2006;48: 1527–37. 10.1016/j.jacc.2006.06.055 17045884

[13] Lyons KS, Vellone E, Lee CS, Cocchieri A, Bidwell JT, D’Agostino F, et al A dyadic approach to managing heart failure with confidence. J Cardiovasc Nurs. 2015;30(45): S64–S71. 25658186 10.1097/JCN.0000000000000234

[14] Agren S, Evangelista LS, Hjelm C, Stromberg A. Dyads affected by chronic heart failure: a randomized study evaluating effects of education and psychosocial support to patients with heart failure and their partners. J Cardiac Fail. 2012;18: 359–66. 10.1016/j.cardfail.2012.01.014 PMC3381875 22555264

[15] Kessing D, Denollet J, Widdershoven J, Kupper N. Psychological determinants of heart failure self-care: systematic review and meta-analysis. Psychosom Med. 2016;78: 412–31. 10.1097/PSY.0000000000000270 27082055

[16] Adams J, Kuchibhatla M, Christopher EJ, Alexander JD, Clary GL, Cuffe MS, et al Association of depression and survival in patients with chronic heart failure over 12 years. Psychosomatics 2012; 53(4): 339–46. 10.1016/j.psym.2011.12.002 22281436 PMC3731067

[17] Lee CS, Suwanno J, Riegel B. The relationship between self-care and health status domains in Thai patients with heart failure. Eur J Cardiovasc Nurs. 2009;8: 259–66. 10.1016/j.ejcnurse.2009.04.002 19411188 PMC2757500

[18] Navidian A, Yaghoubinia, Ganjail A, Khoshsimaee S. The effect of self-care education on the awareness, attitude, and Adherence to self-care behaviours in hospitalised patients due to heart failure with and without depression. PLOS One. 2015 6 19 10.1371/journal.pone.0130973 PMC4475047 26091101

[19] Agren S, Evangelista L, Davidson T, Stromberg A. The influence of chronic heart failure in patient-partner dyads. A comparative study addressing issues of health-related quality of life. J Cardiovasc Nurs. 2011; 26(1): 65–73. 10.1097/JCN.0b013e3181ec0281 21127426 PMC3246077

[20] Hooker SA, Schmiege SJ, Trivedi RB, Amoyal NR, Bekelman DB. Mutuality and heart failure self-care in patients and their informal caregivers. J Cardiovasc Nurs. 2018;17(2): 102–13. 10.1177/1474515117730184 PMC9390005 28868917

[21] Pressler SJ, Gradus-Pizlo I, Chubinski SD, Smith G, Wheeler S, Sloan R, et al Family caregivers of patients with heart failure: a longitudinal study. J Cardiovasc Nurs. 2013;28: 417–28. 10.1097/JCN.0b013e3182563877 22760173

[22] Chung ML, Pressler SJ, Dunbar SB, Lennie TA, Moser DK. Predictors of depressive symptoms in caregivers of patients with heart failure. J Cardiovasc Nurs. 2010;25: 411–19. 10.1097/JCN.0b013e3181d2a58d 20714239 PMC2924771

[23] Chung ML, Lennie TA, Mudd-Martin G, Dunbar SB, Pressler SJ, Moser DK. Depressive symptoms in patients with heart failure negatively affect family caregiver outcomes and quality of life. Eur J Cardiovasc Nurs. 2016;15(1): 30–8. 10.1177/1474515114535329 24829295

[24] Rohrbaugh MJ, Shoham V, Cleary AA, Berman JS, Ewy GA. Health consequences of partner distress in couples coping with heart failure. Heart Lung. 2009;38(4): 298–305. 10.1016/j.hrtlng.2008.10.008 19577701

[25] Hooley PJD, Butler G, Howlett JG. The relationship of quality of life, depression, and caregiver burden in outpatients with congestive heart failure. Congest Heart Fail. 2005; 11(6): 303–10. 10.1111/j.1527-5299.2005.03620.x 16330905

[26] Bidwell JT, Lyons KS, Lee CS. Caregiver well-being and patient outcomes in heart failure: a meta-analysis. J Cardiovas Nurs. 2017;32(4): 372–82. 10.1097/JCN.0000000000000350 PMC5346066 27617564

[27] Agren S, Evangelista LS, Stromberg A. Do partners of patients with heart failure experience caregiver burden? Eur J Cardiovasc Nurs. 2010;9: 254–62. 10.1016/j.ejcnurse.2010.03.001 20598946 PMC3180929

[28] Liljeroos M, Agren S, Jaarsma T, Stromber A. Dialogue between nurses, patients with heart failure and their partners during a dyadic psycho-educational intervention: a qualitative study. BMJ Open 2017;e018236 10.1136/bmjopen-2017-018236 29247098 PMC5736023

[29] Buck HG, Harkness K, Wion R, et al Caregivers’ contributions to health failure self-care: a systematic review. Eur J Cardiovasc Nurs. 2015;14: 79–89. 10.1177/1474515113518434 24399843

[30] Vellone E, Chung ML, Cocchieri A, Rocco G, Alvero R, Reigal B. Effects of self-care on quality of life in adults with heart failure and their spousal caregivers: testing dyadic dynamics using the Actor-Partner Interdependence Model. J Fam Nurs. 2014; 20: 120–41. 10.1177/1074840713510205 24189325

[31] Hooker SA, Grigsby ME, Riegal B, Bekelman DB. The impact of relationship quality on health-related outcomes in heart failure patients and informal family caregivers. An integrative review. J Cardiovasc Nurs. 2015;30: S52–S63. 10.1097/JCN.0000000000000270 25955196

[32] Molloy GJ, Johnston DW, Witham MD: Family caregiving and congestive heart failure. Review and analysis. Eur J Heart Fail. 2005;7: 592–603. 10.1016/j.ejheart.2004.07.008 15921800

[33] Luttik ML, Jaarsma T, Vegger N, Tijssen J, Sanderson R, van Veldhuisen DJ. Caregiver burden in partners of heart failure patients: limited influence of disease severity. European Journal of Heart Failure 2007;9:695–710. 10.1016/j.ejheart.2007.01.006 17347035

[34] Retrum JH, Nowels CT, Bekelman DB. Patient and caregiver congruence. The importance of dyads in heart failure care. J Cardiovasc Nurs. 2013,28(2): 129–136. 10.1097/JCN.0b013e3182435f27 22343213

[35] Lum HL, Lo DL, Hooker SA, et al Caregiving in heart failure: Relationship quality is associated with caregiver benefit finding and caregiver burden. Heart Lung. 2014;43: 306–10. 10.1016/j.hrtlng.2014.05.002 24992881 PMC5711727

[36] Stamp KD, Dunbar SB, Clark PC, Reilly CM, Gary RA, Higgins M, et al Family context influences psychological outcomes of depressive symptoms and emotional quality of life in patients with heart failure. J Cardiovasc Nurs. 2014;29(6): 517–27. 10.1097/JCN.0000000000000097 24434821 PMC4098026

[37] Chung ML, Moser DK, Lennie TA, Rayens MK. The effects of depressive symptoms and anxiety on quality of life in patients with heart failure and their spouses: testing dyadic dynamics using Actor–Partner Interdependence Model. J Psychosom Res. 2009; 67: 29–35. 10.1016/j.jpsychores.2009.01.009 19539816 PMC2732117

[38] Lee KS, Lennie TA, Wu J-R, Biddle MJ, Moser DK. Depressive symptoms, health-related quality of life, and cardiac event-free survival in patients with heart failure: a mediation analysis. Qual Life Res. 2014;23: 1869–76. 10.1007/s11136-014-0636-5 24488573

[39] Goodman H, Firouzi A, Banya W, Lau-Walker M, Cowie MR. Illness perception, self-care behaviour and quality of life of heart failure patients: a longitudinal questionnaire survey. Int J Nurs Stud. 2013;50(7): 945–53. 10.1016/j.ijnurstu.2012.11.007 23211796

[40] Ayotte BJ, Margrett JA, Patrick JH. Dyadic analysis of self-efficacy and perceived support: The relationship of individual and spousal characteristics with physical activity among middle-aged and young-older adults. Psychol Aging. 2013;28(2): 555–63. 10.1037/a0032454 23795767

[41] Kenny DA, Kashy DA and Cook WL. Dyadic data analysis. New York: The Guilford Press; 2006.

[42] Cook WL and Kenny DA. The actor-partner interdependence model: A model of bidirectional effects in developmental studies. Int J Behav Dev. 2005;29(2): 101–09.

[43] Wickham RE and Knee CR. Interdependence theory and the actor-partner interdependence model: Where theory and method converge. Pers Soc Psychol Rev. 2012; 16: 375–93. 10.1177/1088868312447897 22619276

[44] Derogratis LR, Melisaratos N. The Brief Symptom Inventory: an introductory report. Psychol Med. 1983;13: 595–605. 6622612

[45] Derogratis LR. Brief Symptom Inventory: administration, scoring, and procedures manual. 3rd ed, Minneapolis; National Computer Systems; 1993.

[46] Frances VM, Rajan P, Turner N. British community norms for the Brief Symptom Inventory. Brit J Clin Psychol. 1990;29: 115–6. 2310864 10.1111/j.2044-8260.1990.tb00857.x

[47] Ryan C. British outpatient norms for the Brief Symptom Inventory. Psychol Psychother-T. 2007;80: 183–91. 10.1348/147608306X111165 17535593

[48] Derogatis LR, Unger R. Symptom Checklist-90-Revised. The Corsini Encyclopedia of Psychology. Hoboken, NJ: John Wiley & Sons, Inc.; 2010.

[49] Pereda N, Forns M, Maribel Peró M. Dimensional structure of the Brief Symptom Inventory with Spanish college students. Psicothema. 2007;19(4):634–63. 17959119

[50] Wieland J, Wardenaar KJ, Fontein E, Zitman FG. Utility of the Brief Symptom Inventory (BSI) in psychiatric outpatients with intellectual disabilities. J Intell Disabil Res. 2011;56(9):843–53. 10.1111/j.1365-2788.2011.01440.x 21726320

[51] Mohammadkhani P, Dobson KS, Amiri M, Ghafari FH. Psychometric properties of the Brief Symptom Inventory in a sample of recovered Iranian depressed patients. Int J Clin Hlth Psych. 2010;10(3):541–51.

[52] Derogratis LR, Melisaratos N. The Brief Symptom Inventory: an introductory report. Psychol Med. 1983;13: 595–605. 6622612

[53] Kellett S, Beail N, Newman DW, Hawaes A. The factor structure of the Brief Symptom Inventory: intellectual disability evidence. Clin Psychol and Psychot. 2004;11(4):275–81.

[54] Daoud FS, Abojedi AA. Equivalent factorial structure of the Brief Symptom Inventory (BSI) in clinical and nonclinical Jordanian populations. Eur J Psychol Assess. 2010;26: 116–21.

[55] Schwannauer M, Chetwynd P. The Brief Symptom Inventory: A validity study in two independent Scottish samples. Clin Psychol and Psychot. 2007;14(3):221–8.

[56] Loutsiou-Ladd A, Panayiotou G, Kokkinos CM. A review of the factorial structure of the Brief Symptom Inventory (BSI): Greek evidence. Int J Test. 2008;8(1):90–110.

[57] Croog SH, Kong BW, Levine S, Weir MR, Baume RM. Hypertensive black men and women. Arch Intern Med. 1990;150(8):1733–1741. 10.1001/archinte.150.8.1733 2200384

[58] Croog SH, Levine S, Testa MA, Brown B, Bulpitt CJ, Jenkins CD, et al The effects of antihypertensive therapy on the quality of life. New Engl J Med. 1986; 314:1657–64. 10.1056/NEJM198606263142602 3520318

[59] Kushner MG, Beitman BD, Beck NC. Factors predictive of panic disorder in cardiology patients with chest pain and no evidence of coronary artery disease: A cross-validation. J Psychosom Res. 1989; 33(2):207–15. 10.1016/0022-3999(89)90048-2 2724197

[60] Khalil AA, Hall LA, Moser DK, Lennie TA, Frazier SK. The psychometric properties of the Brief Symptom Inventory Depression and Anxiety Subscales in patients with heart failure and with or without renal dysfunction. Arch Psychiat Nurs. 2011; 25(6):419–29. 10.1016/j.apnu.2010.12.005 22114796

[61] Roser LP. Biobehavioral influences of anxiety, depression, and hostility symptoms on health-related outcomes in patients with heart failure [thesis]. Lexington, Kentucky: University of Kentucky; 2016.

[62] Banerjee T, Lee KS, Browning SR, Hopenhayn C, Westneat S, Biddle MJ, et al Limited association between perceived control and health-related quality of life in patients with heart failure. J Cardiovasc Nurs. 2014;29(3): 227–31. 10.1097/JCN.0b013e31828b2b23 23507705 PMC3701030

[63] Dekker RL, Lennie T, Doering LV, Chung ML, Wu J-R, Moser DK. Coexisting anxiety and depressive symptoms in patients with heart failure. Eur J Cardiovasc Nurs. 2014;13(2): 168–76. 10.1177/1474515113519520 24408885 PMC3992982

[64] Vilchinsky N., Dekel R., Leibowitz M., Reges O., Khaskia A., & Mosseri M. Dynamics of support perceptions among couples coping with cardiac illness: The effect on recovery outcomes. Health Psychol. 2011;30(4): 411–9. 10.1037/a0023453 21480711

[65] Easton K, Coventry P, Lovell K, Carter LA, Deaton C. Prevalence and measurement of anxiety in samples of patients with heart failure. J Cardiovasc Nurs. 2016; 31(4): 367–79. 10.1097/JCN.0000000000000265 25930162 PMC4915759

[66] De Jong MJ, Chung ML, Wu-JR. et al Linkages between anxiety and outcomes in heart failure. Heart Lung. 2011;40: 393–404. 10.1016/j.hrtlng.2011.02.002 21453974 PMC3149715

[67] Auld JP, Mudd JO, Gelow JM, Lyons KS, Hiatt SO, Lee CS. Patterns of heart failure symptom are associated with self-care behaviors over 6 months. Eur J Cardiovasc Nurs. 2018;17(6): 543–51. 10.1177/1474515118759074 29442523 PMC6067986

[68] Chung ML, Moser DK, Lennie TA, Rayens MK. The effects of depressive symptoms and anxiety on quality of life in patients with heart failure and their spouses: testing dyadic dynamics using Actor–Partner Interdependence Model. J Psychosom Res. 2009; 67: 29–35. 10.1016/j.jpsychores.2009.01.009 19539816 PMC2732117

[69] Pressler SJ, Gradus-Pizlo I, Chubinski SD, Smith G, Wheeler S, Sloan R, et al Family caregivers of patients with heart failure: a longitudinal study. J Cardiovasc Nurs. 2013;28: 417–28. 10.1097/JCN.0b013e3182563877 22760173

[70] Lee CS, Mudd JO, Auld J, Gelow JM, Hiatt SO, Chien CV, et al Patterns, relevance and predictors of heart failure dyadic symptom appraisal. Eur J Cardiovasc Nurs. 2017;16(7):595–604. 10.1177/1474515117700760 28895484

[71] Rector TS, Cohn JN. Assessment of patient outcome with the Minnesota Living with Heart Failure questionnaire: reliability and validity during a randomized, double-blind, placebo-controlled trial of pimobendan. Pimobendan Multicenter Research Group. Am Heart J. 1992;124: 1017–25. 10.1016/0002-8703(92)90986-6 1529875

[72] Rector TS, Kubo SH, Cohn JN. Validity of the Minnesota Living with Heart failure questionnaire as a measure of therapeutic response to enalapril or placebo. Am J Cardiol. 1993;71(12): 1106–07. 10.1016/0002-9149(93)90582-w 8475878

[73] Scott LD. Caregiving and care receiving among a technologically dependent heart failure population. Adv Nurs Sci. 2000;23: 82–97. 10.1097/00012272-200012000-00008 11104326

[74] Pietri G, Van Ganse E, Ferrer M, Garin O, Wiklund I. MLHF Minnesota Living with Heart Failure Questionnaire. User Manual. MAPI Research Institute; 2004.

[75] Garin O, Ferrer M, Pont A, Wiklund I, Van Ganse E, Vilagut G, et al Evidence on the global measurement model of the Minnesota Living with heart failure questionnaire. Qual Life Res. 2013;22(10): 2675–84. 10.1007/s11136-013-0383-z 23677481

[76] Riegal B, Moser DK, Glaser D, Carlson B, Deaton C, Armola R, et al The Minnesota Living with Heart Failure Questionnaire: sensitivity to differences and responsiveness to intervention intensity in a clinical population. Nurs Res. 2002;51: 209–18. 10.1097/00006199-200207000-00001 12131233

[77] Heo S, Moser DK, Lennie TA, Fischer M, Smith E, Walsh MN. Modifiable correlates of physical symptoms and health-related quality of life in patients with heart failure: a cross-sectional study. Int J Nurs Stud. 2014;51: 1482–90. 10.1016/j.ijnurstu.2014.03.005 24745914

[78] Heo S, Moser DK, Riegel B, Hall LA, Christman N. Testing the Psyhometric properties of the Miinesota Living with heart failure Questionnaire. Nurs Res. 2005;54(4): 265–72. 10.1097/00006199-200507000-00009 16027569

[79] National Statistics. Office of National Statistics. London; 1998.

[80] Carstairs V and Morris R. Deprivation and Health in Scotland. Newcastle-upon-Tyne: Aberdeen University Press; 1991.

[81] Stas L, Kenny DA, Mayer A, Loeys T. Givinh dyadic data analysis away: A user-friendly app for actor-partner interdependence models. Personal Relationships. 2018;25:103–19.

[82] Bidwell JT, Higgins MK, Reilly CM, Clark PC, Dunbar SB. Shared heart failure knowledge and self-care outcomes in patient-caregiver dyads. Heart Lung. 2018; 47(1):32–39. 10.1016/j.hrtlng.2017.11.001 29153759 PMC5722704

[83] Moser DK, Dracup K, Evangelista LS, Zambroski CH, Lennie TA, Chung ML, et al Comparison of prevalence of symptoms of depression, anxiety and hostility in elderly patients with heart failure, myocardial infarction, and coronary artery bypass graft. Heart Lung. 2010;39: 378–85. 10.1016/j.hrtlng.2009.10.017 20561849 PMC2939239

[84] Thomson P, Molloy GJ, Chung ML. The effects of perceived social support on quality of life in patients awaiting coronary artery bypass grafting and their partners: testing dyadic dynamics using the Actor-Partner Interdependence Model. Psychol Health Med. 2012;17(1): 35–46. 10.1080/13548506.2011.579988 21678197

[85] Thomson P, Howie K, Mohan ARM, Chung ML. Evaluating perceptions of self-efficacy and quality of life in patients having coronary artery bypass grafting and their family caregivers. J Cardiovasc Nurs. 2019;34(3): 250–7. 10.1097/JCN.0000000000000553 30489417

[86] Chung ML, Bakas T, Plue LD, Williams LS. Effects of self-esteem, optimism, and perceived control on depressive symptoms in stroke survivor-spouse dyads. J Cardiovasc Nurs. 2016;31(2): 8–16. 10.1097/JCN.0000000000000232 PMC4526460 25658182

[87] Martensson J, Dracup K, Canary C, Fridlund B. Living with heart failure: depression and quality of life in patients and spouses. J Heart Lung Transplant 2003;22(4):460–7. 10.1016/s1053-2498(02)00818-5 12681424

[88] Pihl E, Jacobsson A, Fridlund B, Stromberg A, Martensson J. Depression and health-related quality of life in elderly patients suffering from heart failure and their spouses: a comparative study. Eur J Heart Fail. 2005;7(4): 583–9. 10.1016/j.ejheart.2004.07.016 15921798

[89] Gump B.B., Kulik J.A. Stress, affiliation, and emotional contagion. Journal of Personality & Social Psychology. 1997;72: 305–19. 9107002 10.1037//0022-3514.72.2.305

[90] Neumann R., Strack F. ‘Mood contagion’: transfer of mood between persons. Journal of Personality & Social Psychology. 2000;79:211–223. 10948975 10.1037//0022-3514.79.2.211

[91] Evangelista LS, Dracup K, Doering L, Westlake C, Fonarow GC, Hamilton M. Emotional well-being of heart failure patients and their caregivers. J Card Fail. 2002;8(5): 300–5. 10.1054/jcaf.2002.128005 12411980

[92] Liljeroos M, Stromberg A, Arestedt K, Chung ML. Mediation effect of depressive symptoms in the relationship between perceived control and wellbeing in patients with heart failure and their partners. Eur J Cardiovasc Nurs. 2018;17(6): 527–34. 10.1177/1474515118755721 29381082

[93] Sears SF, Vazquez Sowell LD, Kuhl EA, Kovacs AH, Serber ER, Handberg E, et al The ICD shock and stress management program: a randomised trial of psychosocial treatment to optimise quality of life in ICD patients. Pacing Clin Electrophysiol. 2007;30(7): 858–64. 10.1111/j.1540-8159.2007.00773.x 17584267

[94] Lewin RJ, Coulton S, Frizelle DJ, Kaye G, Cox H. A brief cognitive behavioural pre-implantation and rehabilitation programme for patients receiving an Implantable Cardioverter Defibrillator improves physical health and reduces psychological morbidity and unplanned re-admissions. Heart. 2009;95(1): 63–9. 10.1136/hrt.2007.129890 18070951

